# Multi-Headed Conv-LSTM Network for Heart Rate Estimation during Daily Living Activities

**DOI:** 10.3390/s21155212

**Published:** 2021-07-31

**Authors:** Michał Wilkosz, Agnieszka Szczęsna

**Affiliations:** Department of Computer Graphics, Vision and Digital Systems, Faculty of Automatic Control, Electronics and Computer Science, Silesian University of Technology, Akademicka 16, 44-100 Gliwice, Poland; michwil045@student.polsl.pl

**Keywords:** deep learning, heart rate estimation, PPG signal, wearables

## Abstract

Non-invasive photoplethysmography (PPG) technology was developed to track heart rate during physical activity under free-living conditions. Automated analysis of PPG has made it useful in both clinical and non-clinical applications. Because of their generalization capabilities, deep learning methods can be a major direction in the search for a heart rate estimation solution based on signals from wearable devices. A novel multi-headed convolutional neural network model enriched with long short-term memory cells (MH Conv-LSTM DeepPPG) was proposed for the estimation of heart rate based on signals measured by a wrist-worn wearable device, such as PPG and acceleration signals. For the PPG-DaLiA dataset, the proposed solution improves the performance of previously proposed methods. An experimental approach was used to develop the final network architecture. The average mean absolute error (MAE) of the final solution was 6.28 bpm and Pearson’s correlation coefficient between the estimated and true heart rate values was 0.85.

## 1. Introduction

Current changes in human lifestyle have resulted in a lack of physical activity, which is one of the leading risk factors for non-communicable diseases and deaths [1]. Research demonstrates that an adequate amount of physical activity can significantly contribute to the prevention and control of diseases such as cardiovascular disease, cancer, and diabetes. Awareness of the positive aspects of physical activity, along with progress in the field of microcontrollers, has contributed to the development of many wearable devices that allow for continuous physical activity monitoring. Wearables have become a part of many people’s lives, which has led them to pay more attention to their health by monitoring physical activity [2,3,4,5].

A common feature of many such devices is the ability to monitor continuous heart rate (HR). In most wearables, the measurement is based on photoplethysmography (PPG) signals. PPG signals are obtained through the emitting of light (using a light-emitting diode, LED) on the skin and measuring (using a photodiode) the variations in reflected or transmitted light intensity. The periodicity of measured light corresponds to the cardiac rhythm, which is used for HR estimation [6]. However, this method has one major drawback: during intense physical activity, the PPG signal is very susceptible to interference [7]. Reducing the influence of motion artifacts is currently a leading research direction in HR estimation. The ideal HR estimation method should be able to predict HR under free-living conditions without the need for individual calibration during daily life activities such as sitting, standing, and walking, or while participating in sports activities [8]. Researchers usually solve the described problem through advanced PPG signal processing or by enriching the developed methods with accelerometer signals that directly inform about the movement aspects of the device [9]. In particular, many new methods have been developed based on two IEEE Signal Processing Cup 2015 (ISPC) datasets [10].

Promising classical solutions such as SpaMa [11] and Schaeck2017 [12] achieve very high accuracy for ISPC datasets, with an accuracy of 1–4 beats per minute (bpm), using mean absolute error (MAE) as a metric. During tests on more complex datasets, the versatility of these approaches proved to be limited [13]. Good results were achieved only with session optimization of the used solutions, which makes it impossible to quickly apply these methods to other data. These algorithms have in common the inclusion of several adjustable parameters, the tuning of which, for each dataset-session specifically, has been reported as typically having very low MAE results. Leave-one-session-out (LOSO) cross-validation is considered a universal solution; methods prepared in this way can be applied to current data from everyday life situations. Furthermore, the data used to develop these methods only contain less than one measurement hour and a few physical activities, and were performed under laboratory conditions. Such a small amount of data and conditions that do not correspond to daily life result in very limited possibilities for developed solutions from which to generalize [14].

To overcome this problem, a novel dataset (PPG-DaLiA [13]) was acquired for the HR estimation problem. Classical methods for LOSO cross-validation using the PPG-DaLiA dataset give MAE values of about 15.56 bpm (SpaMa algorithm) and 20.45 bpm (Schaeck2017 algorithm) [13]. A large amount of data in the dataset makes it possible to develop methods using deep learning, such as the Deep PPG solution [13]. The model is based on data from a single PPG channel and a 3-axis accelerometer signal. The data were divided into 8 s time windows with a shift of 2 s. The next step was to apply a fast Fourier transform (FFT) on each time series window. Then, each of the obtained spectra was filtered, leaving only a range from 0–4 Hz. The final preprocessing step was to apply z-score standardization for each channel of the spectrum in an 8 s window. The Deep PPG network has the following configuration for HR prediction:

- 2D convolutional layer,
- 1D convolutional layer followed by max-pooling layer,
- seven 1D convolutional layers, each one followed by a max-pooling layer,
- 1D convolutional layer,
- flattening layer,
- fully connected layer followed by dropout layer,
- fully connected layer.

The goal of the Deep PPG network was to solve the regression task and determine the heart rate value for an 8 s time window. It is possible to treat the time-frequency spectrum as a two-dimensional image, which, after the first 2D convolutional layer, is further treated as a typical time series, and is then processed using 1D convolutional layers. For each training session of the network, for a given set of test data, average values from 7 repetitions are saved, which together form one model. The test results of Deep PPG have demonstrated potential inaccuracy in ensemble prediction.

Another promising solution is also based on deep learning, in which HR estimation was obtained from reflectance PPG with an acceleration power spectrum and acceleration intensity value [15]. The data used included multi-channel PPG and signals from a 3-axis accelerometer. The data were prepared in 8 s windows with a shift of 2 s and were filtered by a fourth-order Butterworth bandpass filter with cutoff frequencies in the range 0.4–4 Hz. Then the signal was standardized using a z-score and downsampled to 25 Hz. After downsampling, a power spectrum was created using an FFT. Each 8 s window went into the model as a time-frequency spectrum and was again standardized using a z-score. The proposed network architecture in this approach consisted of a total of eight layers [15]:

- 2D convolutional layer followed by max-pooling layer and dropout layer,
- 1D convolutional layer followed by max-pooling layer and dropout layer,
- flattening layer,
- concatenation layer,
- fully connected layer,
- two long short-term memory (LSTM) layers, each one followed by dropout layer,
- fully connected layer followed by softmax layer.

The HR estimation is treated as a multi-class classification problem, and based on this fact the softmax layer provides probabilities corresponding to each estimated frequency bin value, covering a true HR value for an 8 s window. In this solution, of particular interest is the use of LSTM cells that capture the temporal dependencies in the feature maps created by the convolutional layers. This solution showed an average absolute error of less than 1.5 bpm. In addition, the study demonstrated that the obtained model estimates HR for even high-intensity exercise. Unfortunately, the solution has only been tested on data acquired under laboratory conditions, which may indicate overly optimistic results. However, the good results for high-intensity exercise obtained by the solution and the concept of using LSTM cells were additional reasons for the further development shown in this work.

Based on the solutions described above and others that were reviewed [9,16], it can be seen that deep learning, because of its generalization capabilities, can be a major direction in the search for an ideal solution for the HR estimation problem.

This work aimed to propose a novel neural network architecture, multi-headed convolutional neural network model enriched with long short-term memory cells (named MH Conv-LSTM DeepPPG), for HR estimation treated as a regression task. The novel deep learning approach was compared to the Deep PPG solution [13], by performing an evaluation using the PPG-DaLiA dataset. To achieve this goal, an experimental approach was used to develop the final network architecture. Apart from proposing a new network model for HR estimation, the novelty of the presented work is the use of signals in the time domain directly, and not the frequency domain. This feature eliminates the need for additional prepossessing of the input signals.

## 2. Materials and Methods

### 2.1. Evaluation

The standard unit for measuring heart rate is beats per minute (bpm). In the heart rate estimation problem, the most frequently repeated metric is mean absolute error (MAE) [9,13,15,16], which was also used in this work (1), with N being the number of windows in the measurement time series and $bpm^{\prime }$ denoting the estimated and bpm reference heart rate value.

(1) $$MAE\left(bpm^{\prime },bpm\right)=\frac{1}{N}\sum_{i=1}^{N}\left|bpm_{i}^{\prime }-bpm_{i}\right|$$

### 2.2. Dataset

The dataset used in this work is the previously mentioned PPG-DaLiA [13] dataset. This abbreviation stands for *PPG dataset for motion compensation and heart rate estimation in daily life activities* (https://ubicomp.eti.uni-siegen.de/home/datasets/sensors19/, accessed on 31 July 2021). The proposed dataset has the following characteristics that determine its usefulness:

- public access to all survey data,
- large amount of data, a total of 36 hours of data with high sampling rates for 15 study participants performing 8 different types of physical activities (work, walking, lunch, driving, cycling, playing football, stairs, sitting),
- sensor data were obtained from commercially available devices,
- activities were carried out in equivalent conditions to everyday life,
- physical diversity among survey participants,
- a reference in the form of heart rate obtained from electrocardiography (ECG), which is considered the gold standard for HR measurement.

One of the devices used to prepare the dataset was the wrist-worn Empatica E4. The wrist is the most common placement for wearable devices; hence, data from this device are further suitable for use in this work.

### 2.3. Dataset Preparation

An important element that an ideal estimation method must satisfy is its universality. To ensure this condition, the models proposed in this work were trained and tested using cross-validation. Since the dataset consists of 15 complete sessions for different participants, a variation of cross-validation was used that was split into training and validation, and the test sets refer to the individual sessions of the participants. The variation used is called leave-one-out cross-validation (LOOCV), and for the available data, each fold in LOOCV consisted of 12 participants making up the training data set, 2 participants being the validation data set, and 1 participant being the test set. On this participant, the performance of the presented solutions was evaluated. Smaller pieces of research related to building the network architecture were evaluated in 5 folds, while the final solution was evaluated in 15 folds.

The most promising solutions process data in the form of a time-frequency spectrum. In the prestudy, we compared the performance of the Deep PPG model using both frequency and time domain data. Based on this study, we found that for some of the participant’s data in the test set, the time domain was preferable, while for others the frequency domain was better. Therefore, our solution uses data in the time domain, for which preprocessing is reduced to the bare minimum. In this work, the final solution used PPG and 3-axis accelerometer signals. Signals measured by the Empatica E4 device were recorded at different frequencies: 32 Hz for 3 signals from the accelerometer and 64 Hz for the PPG signal. Our final model combined these 4 features. After the dataset was divided into each fold, further processing was performed in 3 simple steps:

1 In the first step, the resolution of the PPG signal was reduced using decimation to 32 Hz.
2 In the second step, standardization was performed using a z-score by removing the mean and scaling the features to unit variance. Standardization according to the generally accepted principle was performed using statistics obtained only for the training set. Then, mean and standard deviation from the training set were used to standardize the validation and test sets.
3 In the third step, for all three sets, the features were processed using the sliding window method. The window size and shift were adapted from most previous works on the HR problem.

Therefore, a single window was 8 s long, which corresponds to one feature of datasets of 256 data points (sampling the 32 Hz signal). The next window was created by a shift of 2 s, making 6 s of feature overlap. Finally, the training data corresponding to the individual subjects were fed into the network. The length of the training sets for each fold slightly varied according to the duration of the entire data collection protocol.

### 2.4. Design of Network Architecture

The basic assumption of the neural network architecture being built for this work was to eliminate, as much as possible, the influence of motion artifacts on the obtained results. Therefore, the entire network architecture design process was only concerned with data from the wrist-worn accelerometer. The idea was simple: if the network could successfully estimate HR from the accelerometer alone, it would be more capable of solving the HR problem using data from the accelerometer and additional features.

In the prestudy, hyperparameters related to the training of networks were selected using a validation set. The selected parameters performed best on early versions of the network architecture, which was the reason for their use. All experiments used the same set of hyperparameters as shown in Table 1.

The 5-fold LOOCV was responsible for the versatility of the results of each experiment performed in building the network. The participants constituting the test sets for each fold were also selected to ensure that the tests provided as much information as possible. Therefore, in addition to the results for the participants for which HR did not deviate significantly from the mean (S3, S9, S15), the experiments will also provided information on how the proposed solutions performed for the user with the highest mean HR (S5) and the lowest (S12). Each network considered in this work solves a regression problem by estimating a single HR value for an 8 s window. In determining the final architecture, we tested the impact of various network elements on the performance metric score.

The first experiment conducted tested the number of filters in the first two convolutional layers in the basic network architecture. It was assumed that in the first convolutional layer, there would be *n* filters, while in the second layer it would be equal to 2n. Increasing the number of filters in the subsequent layers of the convolutional neural network (CNN, Conv) is an often repeated procedure that allows the layers to capture more patterns in data. The experiment conducted tested four different sizes of *n* (24, 48, 96, 128) with the same kernel size (k=3). To reduce the dimensionality of the resulting feature maps, each convolution operation was followed by a max-pooling operation. Then, the resulting feature maps were flattened by a flattening layer, from which they were processed by a fully connected layer. The rectified linear unit was used as a default activation function across the network and to avoid overfitting the dropout layer. The network configuration used in this experiment was as follows:

- 1D convolutional layer (number of filters = n, kernel size = k, strides = 1),
- max-pooling layer (pool size = 3, strides = 3),
- 1D convolutional layer (number of filters = 2n, kernel size = k, strides = 1),
- max-pooling layer (pool size = 3, strides = 3),
- flattening layer,
- fully connected layer (number of neurons = 128),
- dropout layer (size = 0.5),
- fully connected layer (number of neurons = 1).

In total, the network was trained 20 times in this experiment, each time changing the training fold or the number of filters in the first two convolutional layers. The resulting mean MAE scores for all test participants are shown in Table 2.

The obtained results obtained varied slightly depending on the number of filters used. For 64 filters in the first layer and 128 filters in the second layer, the best results were achieved, with an average MAE of 20.36 bpm. The parameters obtained from the test could be used as the basis for further development of the network architecture. However, we assumed that a kernel size adjusted from the vast majority of image recognition works may not be sufficient for time-dependent accelerometer signals, which are the basis for the next experiment.

Small kernel size combined with the max pooling operation resulted in more localized feature extraction from the available signals. In the case of signal representation in the form of a time–frequency spectrum, such a solution may be appropriate, because the spectrum can be treated as a two-dimensional image. However, in the case of a one-dimensional signal, a simpler solution was to use a larger kernel size for the convolution operation, which in turn allows the features to be considered in a wider context. In a second experiment on determining the appropriate network architecture, a test was conducted to determine the effect of a larger kernel size (k=12). The same network architecture was used for the experiment, except that the convolutional layers used a kernel size of 12. Again, the network was trained for individual varying numbers of filters and training folds. The results of the experiment are presented in Table 3.

Based on these results, the best number of filters was 96 in the first layer and 192 in the second layer, as this configuration obtained the best results, with an average MAE of 19.32 bpm. For the larger kernel, a performance improvement is evident for each number of filters tested and, consequently, the best configuration of the first two layers was found, which was used for further experiments.

Increasing the depth of a neural network is a popular procedure that is often used in state-of-the-art solutions in many fields [9,17]. Deeper neural networks allow for the network to learn more complex nonlinear relationships. For the network architecture under development, another test was performed to check the impact of adding more convolutional layers to the network. The tests involved adding 1 to 3 additional convolutional layers, giving a maximum of 5 layers in total, given the existing 2 layers in the already existing architecture. Adding more convolutional layers was impossible because the spatial dimension shrinks to 1 with 3 additional layers. The configuration of the additional layers was as follows:

- First additional convolutional layer:
  - 1D convolutional layer (number of filters = 128, kernel size = 3, strides = 1),
  - max-pooling layer (pool size = 2, strides = 2),
  - dropout layer (size = 0.1).
- Second additional convolutional layers:
  - 1D convolutional layer (number of filters = 192, kernel size = 3, strides = 1),
  - max-pooling layer (pool size = 2, strides = 2),
  - dropout layer (size = 0.1),
  - 1D convolutional layer (number of filters = 128, kernel size = 3, strides = 1),
  - max-pooling layer (pool size = 2, strides = 2),
  - dropout layer (size = 0.1).
- Third additional convolutional layers:
  - 1D convolutional layer (number of filters = 192, kernel size = 3, strides = 1),
  - max-pooling layer (pool size = 2, strides = 2),
  - dropout layer (size = 0.1),
  - 1D convolutional layer (number of filters = 128, kernel size = 3, strides = 1),
  - max-pooling layer (pool size = 2, strides = 2),
  - dropout layer (size = 0.1),
  - 1D convolutional layer (number of filters = 64, kernel size = 3, strides = 1),
  - max-pooling layer (pool size = 2, strides = 2),
  - dropout layer (size = 0.1).

Subsequent convolutional layers already used a kernel size of 3 because their task was to extract more local signal dependencies. For each network configuration, the rest of the network was analogous to the first two experiments:

- flattening layer,
- fully connected layer (number of neurons = 128),
- dropout (size = 0.5),
- fully connected layer (number of neurons = 1).

The first two experiments conducted showed that convolutional layers with larger kernels achieve better results. However, for some test participants, a small kernel size was found to be better. To apply simultaneous feature extraction with convolutions using different kernel sizes, a multi-headed architecture was used. The proposed architecture is a combination of the best number of filters obtained for a smaller and a larger kernel. One part of the model extracts features using two convolutional layers (96 and 192 filters) using a kernel size equal to 12, while the other part of the model performs exactly the same operation using another two convolutional layers (64 and 128 filters) but using a kernel size equal to 3. The resulting network from this concept consists of the following layers:

- **head 1**
  - 1D convolutional layer (number of filters = 64, kernel size = 3, strides = 1),
  - max-pooling (pool size = 3, strides = 3),
  - 1D convolutional layer (number of filters = 128, kernel size = 3, strides = 1),
  - max-pooling (pool size = 3, strides = 3),
  - flattening layer,
- **head 2**
  - 1D convolutional layer (number of filters = 96, kernel size = 12, strides = 1),
  - max pooling (pool size = 3, strides = 3),
  - 1D convolutional layer (number of filters = 192, kernel size = 12, strides = 1),
  - max-pooling (pool size = 3, strides = 3),
  - flattening layer,
- concatenate layer (merging two model heads),
- fully connected layer (number of neurons = 128),
- dropout (size = 0.5),
- fully connected layer (number of neurons = 1).

Each parallel part of the network creates its own map of features, which are then combined in the concatenate layer. The resulting features are then further processed by a fully connected layer with 128 neurons, half of which are switched off in the dropout layer. This is achieved by applying the created different combinations of convolutional neural network (CNN) architectures to the available data. In this case, the following results were obtained and are shown in Table 4. L corresponds to the number of additional convolutional layers used.

The results obtained in this experiment indicate that deepening the network architecture used did not have the intended effect. For the additional convolutional layers, the differences in the results obtained were minimal, indicating that the subsequent feature maps did not add any new information to the network. Surprisingly, the multi-headed model gave the best results for most participants.

Next, improvements were possible by increasing the complexity of the network through the use of recurrent layers. The use of long short-term memory (LSTM) cells can enable the capture of temporal dependencies in the changing signals from accelerometers [18,19]. To enable this, the most promising multi-headed CNN model enriched with LSTM cells (MH Conv-LSTM DeepPPG) was proposed. The LSTM cells were added directly after the convolutional layers. This addition allowed the LSTM cells to process the feature maps obtained using the two convolutional layers for each model head. This experiment investigated 3 upgrades to the existing architecture using LSTM cells. The first two architectures only add a single LSTM cell or stacked LSTM cells per head, while the third architecture uses a time-distributed LSTM layer.

In the architecture using single LSTM layers, each head used layers with 128 cells. Signal outputs from two parallel heads were combined in the concatenation layer, and from there there they moved to a fully connected layer of 256, which corresponded to the size of the combined features. For architecture using stacked LSTM layers, subsequent LSTM layers had 256 units each. This assumption was intended to allow for a broader map of features for combination from the two heads. The resulting features were then processed by a fully connected layer of size 512, with the number of neurons in this layer corresponding to the output size of the concatenation layer. Both tested architectures used a dropout layer with a dropout size of 0.5 to regulate the network.

The use of a time-distributed LSTM layer in the last architecture in this test allowed individual convolutional layers to be applied to each temporal slice of the input data. To make this possible, each time window of length 256 data points was further divided into 8 steps of 32 data points for each accelerometer channel. Thus, the convolutional layers did not extract features from the 256 data points of the channel as a whole, but separately for each of the 8 steps of the time window used. By training the network for each fold and architecture considered, the following results were obtained as shown in Table 5.

The biggest disappointment of the experiment was that the model using the time-distributed LSTM layer performed even worse than architectures without LSTM cells. The reason for this may be the lack of significant time dependencies in the single seconds of the window, which explains the use of 8 s windows in other existing solutions to the HR estimation problem. Nevertheless, the experiment was successful, and further research will use the multi-headed model using the best performing single LSTM layers.

The last experiment in the network architecture design process concerned the size of the last fully connected layer. Four different numbers of neurons *S* (128, 256, 512, 1024) were considered. The task of the fully connected layer was to process the final high-level time-dependent features obtained from the LSTM cells. Selecting the right number of neurons in this layer enabled the network to efficiently learn nonlinear combinations of these features. The network architecture used in this experiment was the same as the best MH Conv-LSTM DeepPPG architecture, only the number of neurons in the fully connected layer was changed between tests. Evaluation results of this experiment are shown in Table 6.

For 512 neurons in the fully connected layer, the results for all participants in the experiment were noticeably higher, which prompted the use of this configuration. The size of the fully connected layer was the last experiment conducted to determine the final network architecture. The resulting network architecture determined from numerous experiments was used for the final solution for HR estimation:

- **head 1**
  - 1D convolutional layer (number of filters = 64, kernel size = 3, strides = 1),
  - max pooling layer (pool size = 3, strides = 3),
  - 1D convolutional layer (number of filters = 128, kernel size = 3, strides = 1),
  - max-pooling layer (pool size = 3, strides = 3),
  - LSTM layer (number of cells = 128),
  - flattening layer,
- **head 2**
  - 1D convolutional layer (number of filters = 96, kernel size = 12, strides = 1),
  - max pooling layer (pool size = 3, strides = 3),
  - 1D convolutional layer (number of filters = 192, kernel size = 12, strides = 1),
  - max-pooling layer (pool size = 3, strides = 3),
  - LSTM layer (number of cells = 128),
  - flattening layer,
- concatenate layer (merging two model heads),
- fully connected layer (number of neurons = 512),
- dropout (size = 0.5),
- fully connected layer (number of neurons = 1).

### 2.5. Implementation

All neural network architectures presented in this paper were developed using TensorFlow (version 2.5.0), the deep learning library that is currently most popular. In addition, the following libraries were used: Python 3.7.10, Pandas 1.1.5, Numpy 1.19.5, Sklearn 0.22.2.post1, Keras 2.5.0.

Calculations were performed using the Google Colaboratory (Colab, https://colab.research.google.com/, accessed on 31 July 2021) environment, a cloud service available free of charge. Colab allows writing and execution of arbitrary Python code through the browser, and is especially well suited to machine learning data analysis.

The best solution presented in this paper can be replicated by downloading the notebook that was used in this work. A link to the framework notebook repository is available on request.

## 3. Results

### 3.1. Final MH Conv-LSTM DeepPPG Network Performance

To objectively evaluate the final solution’s performance, the model was trained a total of 15 times, changing each time the participant constituting the test set. For a full evaluation of the proposed architecture, a PPG signal and a 3-axis accelerometer signal measured by a wrist-worn device were used. The HR predictions obtained for each participant were subjected to performance metrics and thus, for the entire collection protocol, individual participants obtained the following scores as shown in Table 7. The lowest errors are apparent for physical activities that do not require increased intensive movement, such as sitting, driving, lunch, or work. For these activities, the model obtains sufficiently good results that do not require significant improvements. Worse results are noticeable for all activities requiring dynamic movement. The most difficult activities for which the model had to predict HR were climbing stairs, table soccer, cycling, and walking. The movement aspects of these activities significantly worsen the readings from the sensors mounted in the wristband. Despite this, for a large number of participants, the predictions for these activities were not a problem for the network used. An example of these are participants S13, S14, and S15, for whom accurate predicted HR results were acquired for almost every activity. The negative movement aspect is most evident for participant S5, as his results for the low movement activities are comparable to the rest of the participants. However, when the model was tasked with predicting HR for this participant during more intensive activities, his performance worsened significantly. In the case of this participant, the disturbed sensor readings were further compounded by tachycardia.

**Figure 1:**
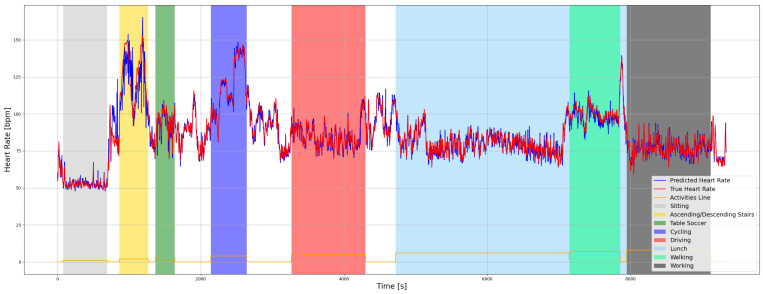
Participant S7 true and predicted HR using proposed MH Conv-LSTM DeepPPG.

**Figure 2:**
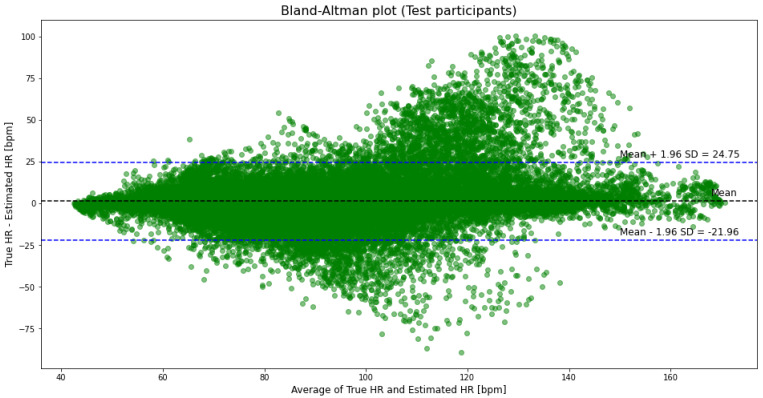
Bland–Altman plot for all participants from PPG-DaLiA dataset. Analysis for all 64,697 HR estimated values, with 3544 (5.48%) points outside the limits of agreement.

**Figure 3:**
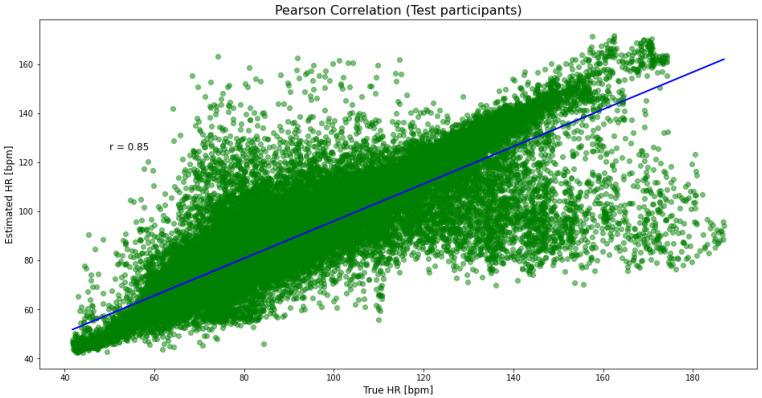
Pearson correlation between estimated and true HR values.

An example of a detailed prediction obtained with the proposed model is shown for participant S7 in Figure 1. Figure 2 presents a Bland–Altman analysis for all participants from the PPG-DaLiA dataset. Pearson’s correlation coefficient is 0.85 (Figure 3) and the *p*-value is <0.05.

### 3.2. Comparison to Deep PPG Solution

The final results obtained were promising, and therefore were compared them with the best solution for the PPG-DaLiA dataset. Both approaches use the same training set size and cross-validation type, so they can be objectively compared. The most significant differences between them, which translate into predictions, are the method of data preparation and the architecture of the network used. Table 8 shows the comparison of the MAE values for each participant between the approach described in this work MH Conv-LSTM DeepPPG and the best model Deep PPG presented in the publication [13]. The solution described in the publication also used ensemble learning to obtain results based on the mean value model weights of 7 repetitions. In the proposed solution, the obtained results were obtained only from a single network training, yet the average MAE score was improved for each test participant. Moreover, the results were obtained after training MH Conv-LSTM DeepPPG for only 100 iterations, with a callback that returns the network weights for which validation loss was the least. There is a colossal difference in favor of the proposed solution when comparing to the Deep PPG that was trained for 15,000 iterations.

**Figure 4:**
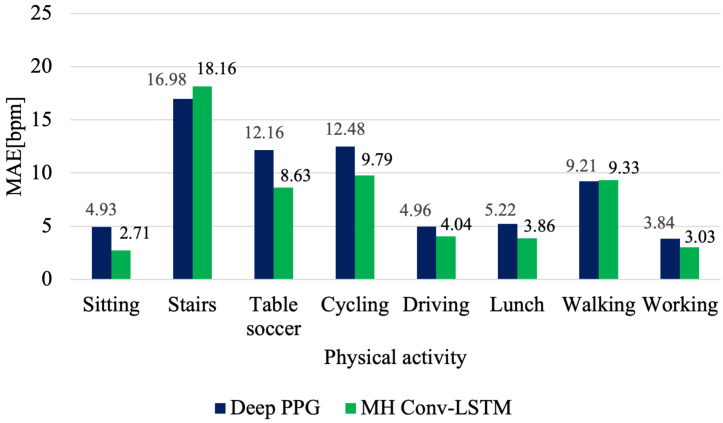
Comparison of the average activity scores for Deep PPG and MH Conv-LSTM DeepPPG. Performance of Deep PPG taken from [13], Table 11.

In the presented solution, the average errors have been improved for the vast majority of activities. The only activities with worse MAE values were climbing stairs and walking. For activities with more dynamic movement, particular improvement is evident for table soccer and cycling, which improved errors by 3.53 bpm and 2.69 bpm, respectively. However, considering the overall model performance, the solution proposed in this work achieves better results (Figure 4).

### 3.3. Results of HR Estimation Using Signals in Frequency Domain

In general, most state-of-the-art solutions to the problem under consideration process data in the form of a time–frequency spectrum. Therefore, the resulting model was tested to see how it handles data in this domain. For this purpose, 3-axis wrist-worn accelerometer signals and a PPG signal were used that were prepared for network training differently than in the other experiments. The two first steps were the same as those used for the other experiments. Firstly, the PPG was downsampled to 32 Hz, and this frequency is the same as that for accelerometer data. Then the signals were processed with a sliding window method with a window size of 8 s and a shift of 2 s. For each time window obtained, an FFT with 2048 points was applied. In the next step, the resulting spectra were truncated, leaving only a 0.4–4 Hz frequency interval. The final step of data preparation was to apply a z-score standardization for each of the windows. The data thus prepared was used to train the model a total of 5 times, each time for a different participant. In this way, the model obtained the following performance metric scores for each tested participant as shown in Table 9.

**Figure 5:**
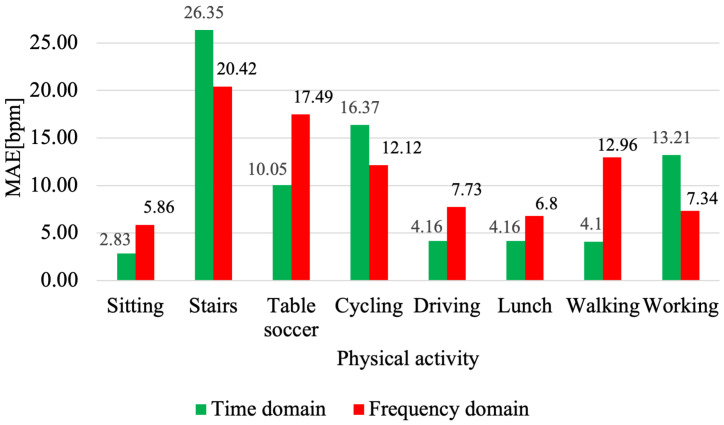
Comparison of average activity scores for different methods of data preparation.

The model was only tested for participants S3, S5, S9, S12, and S15, and the results for these participants show that using the frequency domain gives worse results. Despite this, Figure 5 shows the average MAE results for given participants performing each protocol activity. The frequency domain was found to be better for climbing stairs by 5.93 bpm, cycling by 7.44 bpm, and for work by 5.87 bpm. The results obtained partially explain the comparison with the Deep PPG solution (Figure 5), as the authors also used data in the frequency domain, and as in the Deep PPG solution, climbing stairs is better predicted for this domain in this comparison. The reason for this may be that the time–frequency spectrum focuses on the periodicity of the signals, especially during climbing stairs.

### 3.4. Number of Parameters and Computational Complexity

The proposed model MH Conv-LSTM DeepPPG has about 680K parameters and requires 38M computations for single heart-rate estimation while obtaining lower MAE (6.28 ± 3.53 bpm) values than the model Deep PPG model (named *CNN ensemble* in [13] and achieving MAE value 7.65 ± 4.15 bpm), which is very large and computationally expensive. The model has about 60M parameters and requires 240M computations for single heart-rate estimation. The memory complexity and the number of operations are crucial aspects for the practical realization, especially when it comes to implementation for embedded systems.

In the Colab environment, scripts were running using the following hardware specifications: GPU Tesla K80, 12 GB GDDR5 VRAM; Intel Xeon CPU 2.30 GHz, 13 GB RAM. For HR estimation the GPU utilization was 3% with about 7.14 GB GPU memory and 2.99 GB RAM memory usage.

## 4. Conclusions

The presented results considered a novel neural network model MH Conv-LSTM DeepPPG to estimate HR using data from wearable devices (PPG and 3-axis acceleration signals) and processed directly in the time domain. A detailed analysis of existing solutions allowed the definition of a set of key requirements for the solved problem.

The resulting final architecture, the MH Conv-LSTM DeepPPG network model, was subjected to further evaluation. The HR estimation results obtained have an MAE score equal to 6.28 bpm, which was an improvement over the Deep PPG solution on the PPG-DaLiA dataset by 1.37 bpm (17.9%). The overall results obtained were better for all test participants, but individual predicted activities such as climbing stairs and walking were more favorable for the previous Deep PPG solution. For such activities, an estimation using data in the frequency domain gives better results. Further work is planned to combine estimations based on input signals in the time and frequency domains depending on the parameters of the activity, which may improve the results.

## Figures and Tables

**Table 1:** Hyperparameters related to training.

Hyperparameter	Value/Type
Batch size	128
Learning rate	0.001
Optimizer	Adam
Epochs	100
Loss	Mean squared error

**Table 2:** Results for different numbers of filters with small kernel size (k=3).

Number of Filters	n = 24	n = 48	n = 64	n = 96
**Average MAE (bpm)**	20.93 ± 14.45	20.98 ± 14.11	20.36 ± 14.55	20.43 ± 14.18

**Table 3:** Results for different numbers of filters with large kernel size (k=12).

Number of Filters	n = 24	n = 48	n = 64	n = 96
**Average MAE (bpm)**	20.13 ± 13.52	20.13 ± 13.24	20.10 ± 13.64	19.32 ± 13.93

**Table 4:** Results for different CNN architectures.

CNN Architecture	L = 1	L = 2	L = 3	Multi-Headed
**Average MAE (bpm)**	20.19 ± 14.04	19.17 ± 14.13	19.40 ± 14.32	18.97 ± 14.86

**Table 5:** Results for different MH Conv-LSTM DeepPPG architectures.

MH Conv-LSTM DeepPPG	Single	Stacked	Single Time Distributed
**Average MAE (bpm)**	17.73 ± 13.11	18.73 ± 12.98	20.44 ± 13.03

**Table 6:** Results for different fully connected layer sizes.

MH Conv-LSTM DeepPPG	S = 128	S = 256	S = 512	S = 1024
**Average MAE (bpm)**	17.99 ± 12.03	18.13 ± 11.98	16.88 ± 10.43	19.19 ± 13.31

**Table 7:** MAE (bpm) scores for particular physical activities.

Participant	Sitting	Stairs	Table soccer	Cycling	Driving	Lunch	Walking	Working
S1	4.44	15.05	9.21	4.06	3.32	4.02	5.87	1.83
S2	3.66	17.58	8.01	9.60	4.51	4.50	7.43	4.03
S3	1.94	11.62	4.67	3.81	3.03	3.42	2.84	2.57
S4	2.54	17.42	7.41	9.68	4.70	4.81	8.00	3.85
S5	1.16	66.42	21.40	59.67	2.66	3.87	23.20	3.18
S6	2.34	20.54	16.90	8.40	4.46	-	-	-
S7	1.60	11.25	3.71	2.90	2.12	2.37	2.34	2.03
S8	5.18	17.51	8.05	10.76	8.82	7.97	13.80	5.00
S9	3.74	20.99	5.58	7.93	5.66	5.70	16.19	7.56
S10	1.74	11.21	5.88	5.42	1.72	2.23	2.70	1.99
S11	1.58	19.13	10.54	3.10	3.14	4.77	12.76	1.91
S12	5.77	18.56	13.79	5.52	5.73	5.23	19.17	4.30
S13	2.55	6.87	5.10	5.62	2.54	2.76	4.59	1.61
S14	0.90	4.09	4.41	5.53	4.47	3.96	16.42	3.13
S15	1.54	14.15	4.82	4.90	3.70	2.28	4.65	2.49
**Average**	**2.71 ± 1.50**	**18.16 ± 14.23**	**8.63 ± 5.11**	**9.79 ± 14.02**	**4.04 ± 1.79**	**3.86 ± 1.57**	**9.33 ± 6.84**	**3.03 ± 1.63**

**Table 8:** Comparison of model performance, MAE (bpm). Performance of Deep PPG taken from [13], Table 10.

Participant	Deep PPG	MH Conv-LSTM DeepPPG
S1	7.73	5.13
S2	6.74	4.78
S3	4.03	3.65
S4	5.90	5.61
S5	18.51	16.81
S6	12.88	7.19
S7	3.91	2.88
S8	10.87	9.00
S9	8.79	8.13
S10	4.03	3.99
S11	9.22	6.21
S12	9.35	9.07
S13	4.29	3.45
S14	4.37	4.30
S15	4.17	4.13
**Average**	**7.65 ± 4.15**	**6.28 ± 3.53**

**Table 9:** Metric scores for frequency domain data, MAE (bpm).

Participant	MAE [bmp]
S3	4.52
S5	16.12
S9	9.78
S12	13.42
S15	6.20
**Average**	**10.01**

## Data Availability

The dataset PPG-DaLiA [13] is available on the website https://ubicomp.eti.uni-siegen.de/home/datasets/sensors19/ (accessed on 31 July 2021).
